# Phase II Study of Adjuvant Immunotherapy with the CSF-470 Vaccine Plus Bacillus Calmette–Guerin Plus Recombinant Human Granulocyte Macrophage-Colony Stimulating Factor vs Medium-Dose Interferon Alpha 2B in Stages IIB, IIC, and III Cutaneous Melanoma Patients: A Single Institution, Randomized Study

**DOI:** 10.3389/fimmu.2017.00625

**Published:** 2017-05-31

**Authors:** José Mordoh, María Betina Pampena, Mariana Aris, Paula Alejandra Blanco, Mónica Lombardo, Erika María von Euw, Soledad Mac Keon, Michelle Yépez Crow, Alicia Inés Bravo, Juan Manuel O’Connor, Ana Gabriela Orlando, Franco Ramello, Estrella Mariel Levy, María Marcela Barrio

**Affiliations:** ^1^Instituto Médico Especializado Alexander Fleming, Buenos Aires, Argentina; ^2^Centro de Investigaciones Oncológicas-Fundación Cáncer, Buenos Aires, Argentina; ^3^Fundación Instituto Leloir, IIBBA-CONICET, Buenos Aires, Argentina; ^4^Nobeltri, Buenos Aires, Argentina; ^5^UCLA JCCC-Translational Oncology Research Labs, Los Angeles, CA, United States; ^6^Unidad de Inmunopatología, Hospital Interzonal General de Agudos Eva Perón, San Martín, Argentina; ^7^Hospital Central Olga Rizzi, Reconquista, Argentina; ^8^Instituto Oncológico Mater Dei, Reconquista, Argentina; ^9^Centro Médico San Lucas, Gualeguaychú, Argentina

**Keywords:** cutaneous melanoma, CSF-470 allogeneic cell vaccine, Bacillus Calmette–Guerin, rhGM-CSF, IFN-α2b, Phase II clinical study

## Abstract

The irradiated, allogeneic, cellular CSF-470 vaccine plus Bacillus Calmette–Guerin (BCG) and recombinant human granulocyte macrophage-colony stimulating factor (rhGM-CSF) is being tested against medium-dose IFN-α2b in stages IIB–III cutaneous melanoma (CM) patients (pts) after surgery in an open, randomized, Phase II/III study. We present the results of the Phase II part of the ongoing CASVAC-0401 study (ClinicalTrials.gov: NCT01729663). Thirty-one pts were randomized to the CSF-470 vaccine (*n* = 20) or to the IFN-α2b arm (*n* = 11). During the 2-year treatment, immunized pts should receive 13 vaccinations. On day 1 of each visit, 1.6 × 10^7^ irradiated CSF-470 cells plus 10^6^ colony-forming units BCG plus 100 µg rhGM-CSF were administered intradermally, followed on days 2–4 by 100 µg rhGM-CSF. IFN-α2b pts should receive 10 million units (MU)/day/5 days a week for 4 weeks; then 5 MU thrice weekly for 23 months. Toxicity and quality of life (QOL) were evaluated at each visit. With a mean and a maximum follow-up of 39.4 and 83 months, respectively, a significant benefit in the distant metastasis-free survival (DMFS) for CSF-470 was observed (*p* = 0.022). Immune monitoring showed an increase in antitumoral cellular and humoral response in vaccinated pts. CSF-470 was well tolerated; 20/20 pts presented grades 1–2 dermic reactions at the vaccination site; 3/20 pts presented grade 3 allergic reactions. Other adverse events (AEs) were grade 1. Pts in the IFN-α2b arm presented grades 2–3 hematological (7/11), hepatic (2/11), and cardiac (1/11) toxicity; AEs in 9/11 pts forced treatment interruptions. QOL was significantly superior in the vaccine arm (*p* < 0.0001). Our results suggest that CSF-470 vaccine plus BCG plus GM-CSF can significantly prolong, with lower toxicity, the DMFS of high-risk CM pts with respect to medium-dose IFN-α2b. The continuation of a Phase III part of the CASVAC-0401 study is encouraged.

## Introduction

Despite recent advances in its treatment, cutaneous melanoma (CM), an immunogenic and highly mutated tumor (1, 2), continues to be a dreadful disease. The progressive acquisition of mutations in driver genes (3) confers survival advantage to melanoma cells, but at the same time creates foreign antigens (Ags), the so-called neoantigens or neoepitopes, which would render neoplastic cells detectable by the immune system and target them for destruction (4). Cancer immunotherapies include approaches ranging from stimulating immune effectors to counteracting inhibitory and suppressive mechanisms. Strategies to activate effector immune cells include vaccination with tumor Ags or augmentation of Ag presentation to increase the ability of the patient’s immune system to mount an immune response against neoplastic cells (5). Targeting the immune checkpoints CTLA-4 and PD-1 with monoclonal antibodies (MAbs) aim to release natural breaks that control immune function in physiological situations, to increase and sustain antitumor immune responses, either to shared tumor Ags (i.e., cancer testis Ags) and/or to neoantigens (6). However, although cancer immunotherapy is currently brighter than ever before, the outcome of most patients (pts) remains dismal. The mechanism of action of anti-PD-1 and anti-CTLA-4 MAbs is to release inhibition on CD4 or CD8 T lymphocytes, which recognize tumor-derived peptides restrictively presented in the context of human leukocyte antigen (HLA) molecules. This approach, however, leaves HLA-negative tumor cells unattended, and it has been known for long that HLA downexpression is an escape mechanism for tumor cells (7, 8). To increase the chances of longer survival and even cure, great attention is being focused on adjuvant treatments to pts who, after elimination of macroscopic disease with surgery, might still lodge microscopic tumor foci with a high degree of biological heterogeneity. Present treatment at the adjuvant setting is unsatisfactory. Adjuvant IFN-α2b increases disease-free survival (DFS) but not overall survival (OS); it is accompanied with considerable toxicity (9, 10); and is not universally considered as a gold standard treatment (11). Besides, the optimal dose and duration of IFN-α2b treatment are still unclear (12, 13). A report of the long-term results of the randomized Phase III Trial EORTC 18991 of adjuvant therapy with pegylated-IFN-α2b vs observation in resected stage III CM also concluded that no significant increase in distant metastasis-free survival (DMFS) or OS was noted in the overall population, although some benefit was found in pts with ulcerated tumors (10). Also, adjuvant treatment with ipilimumab has yielded similar results to IFN-α2b, increasing DFS at the expense of considerable immune-related toxicities (14), and a Phase III study comparing both treatments is underway (ECOG 1609). Another randomized study (SWOG S1404) is also currently comparing high-dose IFN-α2b to pembrolizumab in pts at high risk for recurrence and death after surgery (15). Very recently, a Phase III trial (EORTC 18071) of ipilimumab as adjuvant therapy for high-risk stage III melanoma pts, at a dose of 10 mg/kg for 3 years resulted in a significantly higher rate of 5-year recurrence-free survival (40.8%), DMFS (48.3%), and OS (65.4%) than placebo (30.3%, 38.9%, and 54.4% respectively). However, the rate of immune-related toxicities with ipilimumab was substantial and led to the discontinuation of treatment in ~40% of the pts (16).

In this scenario, active immunization with vaccines can be revisited as an interesting treatment, particularly in the adjuvant setting. The interest in cancer vaccination has emerged from the results of several clinical studies demonstrating that vaccines could improve clinical outcome of immunotherapy protocols. Among them we can mention the addition of a peptide vaccine to high-dose interleukin-2 (IL-2) (17), the use of a GM-CSF secreting tumor vaccine in combination with CTLA-4 blockade for metastatic prostate cancer (18), and the use of an autologous vaccine plus Bacillus Calmette–Guerin (BCG) that showed an increased response rate with subsequent ipilimumab for progressive disease in stage III melanoma pts (19). The experience acquired in recent years led to the increasing understanding that cancer immunotherapy is a multifaceted strategy and that combination modalities should be more efficient to prevent disease recurrence.

With the aim of inducing adaptive and innate immune responses against micrometastatic foci, targeting both HLA-positive and HLA-negative tumor cells, we developed the CSF-470 vaccine (Vaccimel), an inert scaffold of four lethally irradiated allogeneic CM cell lines, coadjuvated with BCG and recombinant human granulocyte macrophage-colony stimulating factor (rhGM-CSF). The rationale of such combination is: (i) to use a cytokine-treated, irradiated allogeneic vaccine to challenge the immune system with a broad repertoire of CM Ags (20) and with non-identical HLAs to trigger host-vs-graft reaction; (ii) to use BCG in every vaccination to induce local inflammation, to polarize immune cells toward a Th1 response, and to activate NK cells cytotoxicity and memory-like response (21, 22); (iii) to inject rhGM-CSF with every vaccination, since at low doses this cytokine is a strong monocyte attractant (23, 24), and it is essential to differentiate monocytes into dendritic cells (DCs) (25). It could thus be assumed that this combination should favor Ag uptake by macrophages and DC and boost adaptive immunity through Ag presentation to naïve lymphocytes, either locally or after migration to lymph nodes (26, 27). Besides, it should also increase the innate immunity response. Another Phase III clinical assay using irradiated allogeneic tumor cells (Canvaxin) plus BCG vs placebo with BCG was interrupted due to lack of efficacy. However, in that study, allogeneic cells were not pretreated with cytokines, BCG was only injected twice, and no GM-CSF was used (28).

In a previous Phase I study of Vaccimel plus BCG and escalating doses of rhGM-CSF on 20 pts with stages IIB–IV CM, it was found that 400 µg rhGM-CSF per vaccination divided into four daily doses was optimal (29). From that basis, the Phase II–III trial CASVAC-0401, in which stages IIB, IIC, and III CM pts were randomized to receive the CSF-470 vaccine plus BCG plus GM-CSF vs medium-dose IFN-α2b, was launched in 2009. The results corresponding to the end of the Phase II stage of this study are herein reported.

## Materials and Methods

### CSF-470 Vaccine

The CSF-470 vaccine consists of 1.6 × 10^7^ lethally irradiated cells derived from four CM cell lines established *in-house* (30), MEL-XY1, MEL-XY2, MEL-XY3, and MEL-XX4, which are deposited at the German Collection of Microorganisms and Cell Cultures (DSMZ), Braunschweig, Germany. All cell lines were established from metastatic CM tumors. Cells were cultured as previously described (30). The cell lines were grown in a GMP core facility at the Centro de Investigaciones Oncologicas-FUCA. For vaccine preparation, the four cell lines were thawed, washed, mixed, and subsequently irradiated at 70 Gy (Siemens Lineal Accelerator). The cells were frozen in vaccine doses (1.6 × 10^7^ total cells) in liquid nitrogen until use. For vaccine administration each dose was thawed, washed, and resuspended in Dulbecco’s Modified Eagle Medium.

**Figure 1:**
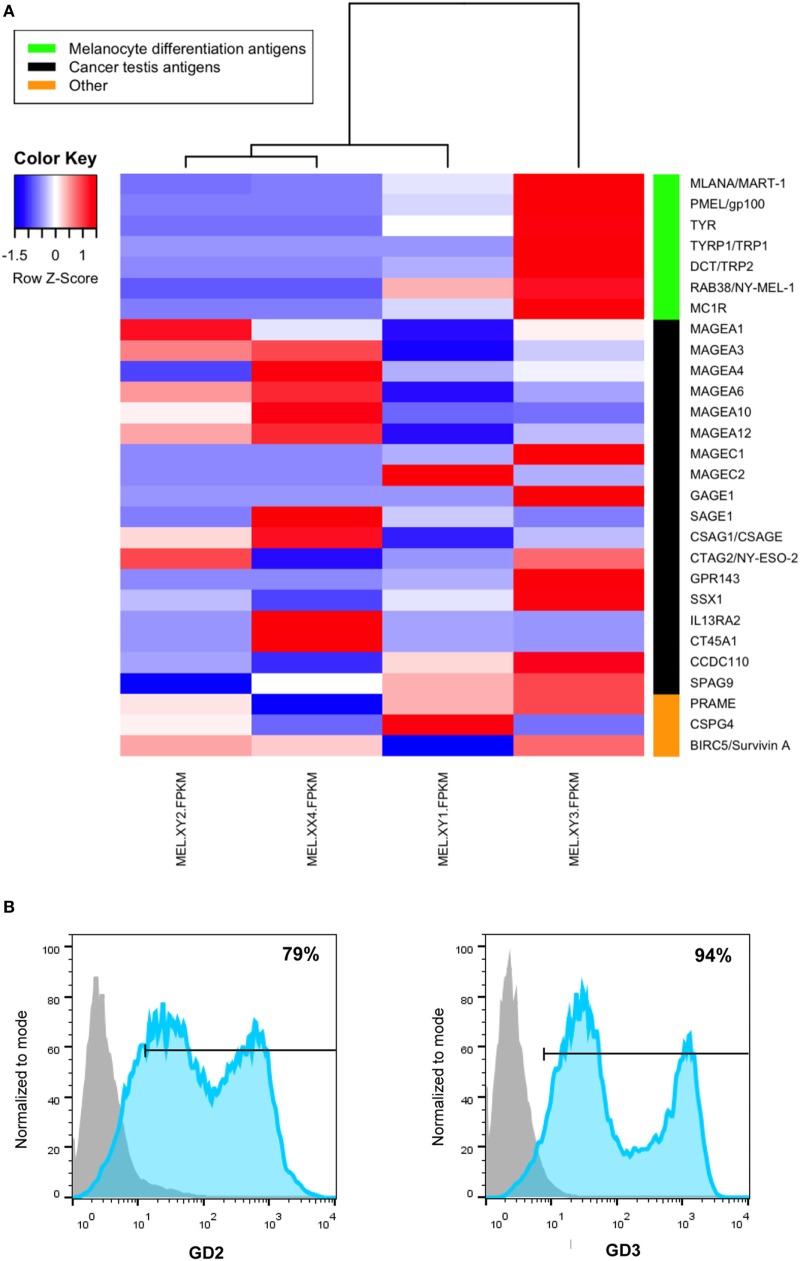
**Antigenic profile of cutaneous melanoma (CM) cell lines that compose the CSF-470 vaccine as determined by RNAseq (A)**. Expression of GD2 and GD3 gangliosides in CSF-470 vaccine as determined by flow cytometry **(B)**; isotype-matched immunoglobulins were used as controls to set unspecific labeling.

RNASeq was performed for each cell line with the sequencing platform Illumina Hiseq 4000, with more than 20 M high-quality single-end reads per sample (BGI Americas). Quality control of reads was performed with FASTX-Toolkit (31) and FastQC (32). Reads were aligned to the latest human Hg38 reference genome using the STAR spliced read aligner (33). Fragment counts were derived using HTSeq package (34). Differentially expressed genes were identified by a ranking based on adjusted *p*-values ≤ 0.005 and a false discovery rate ≤ 0.1 using the R/Bioconductor package edgeR (35). In Figure 1A, we show the expression profile of several melanoma-associated Ags, selected from RNASeq data that have been previously shown to be immunogenic in CM pts (36). Also, vaccine cells express GD2 and GD3 gangliosides as detected by flow cytometry as previously described (37) (Figure 1B).

### Study Design and Treatment

CASVAC-0401 is a single institution, randomized, open label Phase II/III study, to investigate in CM pts stages IIB, IIC, and III postsurgery (adjuvancy), the efficacy and safety (primary objectives) as well as the quality of life (QOL) and immune response (secondary objectives) of the CSF-470 vaccine vs medium-dose IFN-α2b treatment (ClinicalTrials.gov: NCT01729663). A total of 108 pts will be enrolled in the Phase II–III study. As of March 2016, 47 pts were evaluated, of which 31 were eligible and randomized in a 2:1 ratio (vaccine:IFN-α2b). The randomization scheme was generated by using the Web site Randomization.com (http://www.randomization.com); Nobeltri S.R.L. (C.R.O.) was responsible for pts randomization. The study was approved by the Ethics Committee of the Instituto Médico Especializado Alexander Fleming (Buenos Aires, Argentina) and by the Argentine Regulatory Agency (ANMAT), and it is being conducted according to the Declaration of Helsinki Principles. All pts signed informed consent.

**Figure 2:**
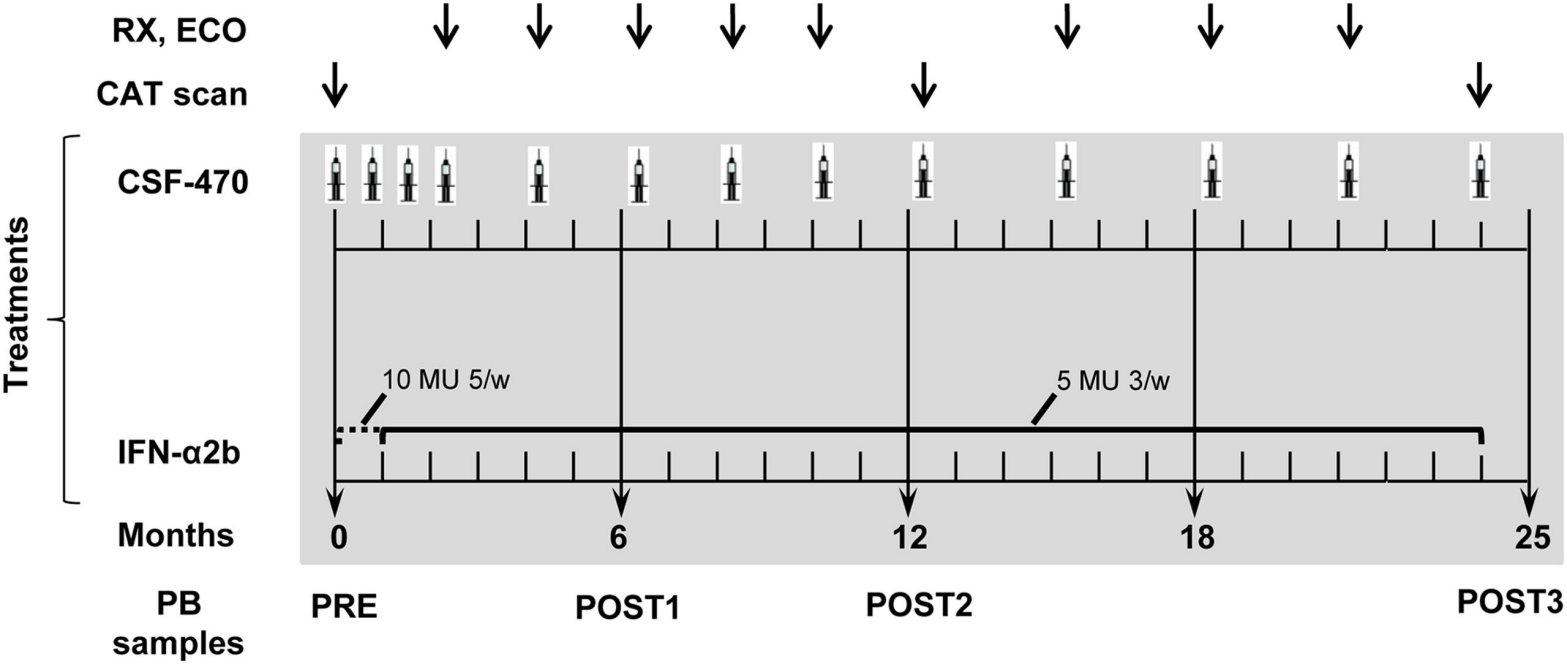
**Randomized Phase II CASVAC-0401 study diagram**.

Vaccine was injected intradermally in the arms or thighs without lymphadenectomy. On day 1, pts received CSF-470 admixed with 1 × 10^6^ colony forming units BCG (Pasteur strain, Instituto Malbrán, Buenos Aires, Argentina) and 100 µg rhGM-CSF (Laboratorio Pablo Cassará, Argentina). On days 2–4, 100 µg rhGM-CSF/day was i.d. injected at the edematous vaccination site. During the 2-year study, pts in the vaccine arm should receive 13 vaccinations: 4 vaccinations 3 weeks apart; 5 vaccinations 2 months apart during the first year and 4 vaccinations 3 months apart during the second year. The pts assigned to the IFN-α2b arm received 10 million units (MU) IFN-α2b (Laboratorio Pablo Cassará, Argentina)/day/5 days a week for 4 weeks; then 5 MU thrice weekly for 23 months (Figure 2).

### Patients

Enrollment conditions were as follows: (a) age between 18 and 65 years; (b) CM stages IIB, IIC, or III (AJCC); (c) *Performance status* Eastern Cooperative Oncology Group (ECOG) 0–1; (d) life expectancy >6 months; (e) no previous chemotherapy or immunotherapy; (f) less than 4 months after surgery; (g) non-evident disease as asserted by CT scans; (h) adequate hematological, hepatic, and renal function; (i) absence of pregnancy, with serum β-HCG determined before each vaccination in premenopausal women; (j) negative serology for HIV, hepatitis B, and hepatitis C. Exclusion criteria were as follows: (a) Type I or II diabetes; (b) pregnancy; (c) morbid obesity; (d) autoimmune diseases or hepatitis; (e) chronic medication with corticosteroids or NSAIDs; and (f) psychiatric disorders. More information about the CASVAC-0401 study is presented in File S1 in Supplementary Material.

### Assessment of Response and Adverse Events (AEs)

During the study, pts should complete 15 visits; visit 1 was for pt selection and visit 15 for closure participation. Visits 2–14 lasted 4 days for CSF-470 pts and 1 day for IFN-α2b pts. At each visit, after evaluation of laboratory results, pts received a complete clinical examination. Imaging studies were performed as indicated in Figure 2. At baseline (PRE) and after 6 (POST1), 12 (POST2), and 24 months (POST3) of treatment, 200 ml of blood were obtained to monitor immunological parameters (Figure 2). AEs were graded and recorded according to the NCI Common Terminology Criteria for Adverse Events, version 3.0. Clinical evaluation: the end points of the study were DMFS, DFS, OS, toxicity, QOL, and immune response.

### Delayed-Type Hypersensitivity (DTH)

On the vaccination day, DTH was performed in the forearm with 1/10th of the CSF-470 dose. The reaction was measured at 1, 24, 48, and 72 h and recorded as follows: 0: macular erythema < 0.5 cm diameter; 1: macular erythema 0.5–1.0 cm; 2: macular erythema 1.1–2.0 cm; 3: macular erythema > 2.0 cm; and 4: papular erythema > 2.0 cm. A DTH score corresponding to the sum of the four values was calculated for each vaccination.

### Immune Populations Profile in Peripheral Blood Mononuclear Cells (PBMC) Samples

Peripheral blood mononuclear cells obtained by density gradient were incubated with anti-human MAbs: PerCP-CD3 (clone SK7), FITC-CD4 (clone RPA-T4), FITC-CD8 (clone RPA-T8), FITC-CD3 (clone UCHT1), and APC-CD56 (clone B159) (BD Biosciences, San Jose, CA, USA). For detection of Tregs, 1 × 10^6^ PBMC were stained with mAbs [FITC-CD4, AF647-FoxP3 (clone 259D/C7) and PE-CD25 (clone M-A251) (BD Biosciences)] following the manufacturer’s protocol. Lymphocytes were gated in FSC/SSC dot plot (≥30,000 events), and NK (CD3^−^CD56^+^), CD4^+^, CD8^+^, and Tregs (CD4^+^ CD25^+^ FoxP3^+^) cells were determined. Isotype-matched irrelevant MAbs were used as negative controls.

NK cells were tested for degranulating activity (CD107a membrane expression in the CD3^−^CD56^+^ population) by flow cytometry, using canonical K562 cells (ATCC, CCL-243) (38) or MelC (non-irradiated CM cells composing CSF-470 vaccine: MEL-XY1, MEL-XY2, MEL-XY3, and MEL-XX4 cell lines) as targets in a 10:1 ratio as previously described (39). All samples were acquired on a BD FACSCalibur using Cellquest Pro software (BD Biosciences, Franklin Lakes, NJ, USA) and analyzed with FlowJo 7.6.2 software (FlowJo, Ashland, OR, USA).

Frequency of myeloid-derived suppressor cells (CD14^+^CD3^−^CD20^−^CD56^−^HLA-DR^−^) was analyzed by flow cytometry. The following mAbs (BD Biosciences) were used: PE-CD14, FITC-CD3, FITC-CD20, APC-CD56, and PerCPCy5.5-HLA-DR (Mouse IgG2a isotype). Data acquisition (≥10,000 events) was performed using a FACSCanto II cytometer, using FACSDiva software (BD Biosciences). Data analysis was performed with FlowJo software.

### IFN-γ Enzyme-Linked Immunospot Assay (ELISPOT)

PRE and POST1 pts’s PBMC and healthy donor (HD) PBMC were thawed, allowed to rest for 2 h at 37°C and then seeded (1 × 10^6^) in 1 ml of Complete Medium consisting of RPMI 1640 (Invitrogen) supplemented with 10% heat-inactivated human AB sera, 2 mM glutamine, 100 U/mL penicillin, 100 µg/ml streptomycin, 2.5 mM HEPES, and 50 U/mL of IL-2 (Laboratorio Pablo Cassará SRL), in 24-well plates (Costar, Corning, NY, USA). PBMC were stimulated with CSF-470 lysate in a 3:1 ratio (presenting cells from PBMC population: lysed CSF-470 cells), and cultured at 37°C, in 5% CO_2_ for 12 days. Every 3 days, fresh complete medium with IL-2 was added. CSF-470 lysate was obtained after five cycles of freezing (liquid nitrogen, 3 min) and thawing (7 min, 37°C) and then centrifuged at 1,000 × *g* for 10 min to separate nuclei. Lysate aliquots were frozen at −80°C until use.

MultiScreen-IP opaque 96-well plates (High Protein Binding Immobilon-P membrane, Millipore, Bedford, MA, USA) were coated overnight at room temperature with 100 μl/well of 5 µg/ml mouse anti-human IFN-γ mAb (BD Biosciences) in 1X PBS (Gibco, ThermoFisher, USA). Then, the plates were washed and blocked for 2 h at room temperature with 200 µl/well of culture medium consisting of RPMI 1640 (Invitrogen) supplemented with 10% heat-inactivated fetal bovine sera (FBS, Gibco), 2 mM glutamine, 100 U/mL penicillin, 100 µg/ml streptomycin, and 2.5 mM HEPES.

Harvested PBMC from 12-day culture from pts (PRE and POST1 samples) and HD were added to the plates in duplicate at 2.5 × 10^5^ cells/well and cultured with either culture medium alone or with addition of OKT3 (30 ng/ml, BD Pharmingen, San Diego, CA, USA) plus PHA (1/1,000, M form, Gibco) for 24 h at 37°C, 5% CO_2_. The plate was washed twice with deionized water and then thrice with PBS–0.05% Tween-20. A total of 1,000 μl/well of 2 µg/ml anti-human IFN-γ-biotinylated mAb (BD Pharmingen) in 1X PBS/10% FBS were added, and the plates were incubated for 2 h at room temperature. After three washes with PBS–0.05% Tween-20, the plate was incubated with 50 μl/well of streptavidin HRP (BD Pharmingen) diluted 1:100 in PBS 1X/10% FBS for 1 h at room temperature. The plates were then washed four times with PBS–0.05% Tween-20 and then twice with PBS. Spots were visualized by adding 50 μl/well of AEC Substrate (BD Biosciences) for 2 min. Substrate reaction was stopped by washing the plate with deionized water. Plates were scanned using an AID *i*SPOT ELR088IFL analyzer to quantify the number of spots per well.

### Monitoring of Humoral Immune Response

Humoral responses were measured against MelC. For pt #006, her own CM cells were obtained from an excised subcutaneous metastasis and grown as a xenograft in NIH nude mice, from which a cell line was established. CM cells were seeded in 96-well culture plates (10^4^ cells/well) and cultured for 48 h. Reactivity was detected by ELISA in PRE, POST1, POST2, and POST3 serum samples (1/10 to 1/1,000 dilutions) using peroxidase-conjugated goat anti-human IgG (Abcam) and *o*-phenylenediamine as substrate (450 nm). A HD serum sample was used to set the background value. In the case of pt #006 cells, a serum sample obtained 2 years after finishing the clinical trial [2 years follow-up (F-UP)] was also analyzed. Pt #006 cells were also incubated with 1/10 diluted sera and bound antibodies (Abs) were detected using FITC-rabbit anti-IgA, G, M (DAKO) and observed by fluorescence microscopy (Olympus BX40 microscope, DP2-BSW software).

### Histopathological and Immunohistochemical Analysis of Tumor Biopsies

Formalin-fixed, paraffin-embedded CM tissue biopsies were studied. Histopathological features were determined according to AJCC-UICC staging (40) (Table S1 in Supplementary Material). Proliferative index (PI) was determined by Ki-67^+^ staining (41) (%): Ki-67^+^ tumor cells/(Ki-67^+^ tumor cells + Ki-67^−^ tumor cells) × 100. PI was determined in 1 mm^2^ tumor hot spot zone (clone MIB-1, Dako). HLA-I expression (%) was determined in tumor tissues with clone EMR8-5 (Abcam). The avidin–biotin–peroxidase (ABC) system (Vectastain, Vector Labs) was afterward used. Sections were examined by optical microscopy (Olympus BX40 microscope, DP2-BSW software), and digitalized pictures were analyzed with ImageJ software (NIH).

### BRAF Status

It was determined after DNA extraction from tumor biopsies, PCR amplification, and Sanger sequencing (42).

### QOL Evaluation

Quality of life was evaluated by the European Organization for Research and Treatment of Cancer Quality of Life Questionnaire (“EORTC QLQ-C30, version 3.0”). Mean scores were calculated for each visit, only considering values from pts who were actually receiving treatment. ECOG performance status was also registered at each visit.

### Statistics

GraphPad Prism statistics software was used. Distribution of pts in both arms of treatment according to different characteristics of pts and tumors was assessed by χ^2^ or *t*-tests. Time to event distribution was estimated by the Kaplan–Meier method. The significance was evaluated by the log-rank test (Gehan–Breslow–Wilcoxon test). For immune monitoring comparisons, paired *t*-tests were used. For DTH and tumor-associated immune populations analysis, the Mann–Whitney test was used. For QOL, unpaired *t*-test with Welch’s correction was used. The significance level was set as *p* < 0.05.

## Results

### Patients

**Figure 3:**
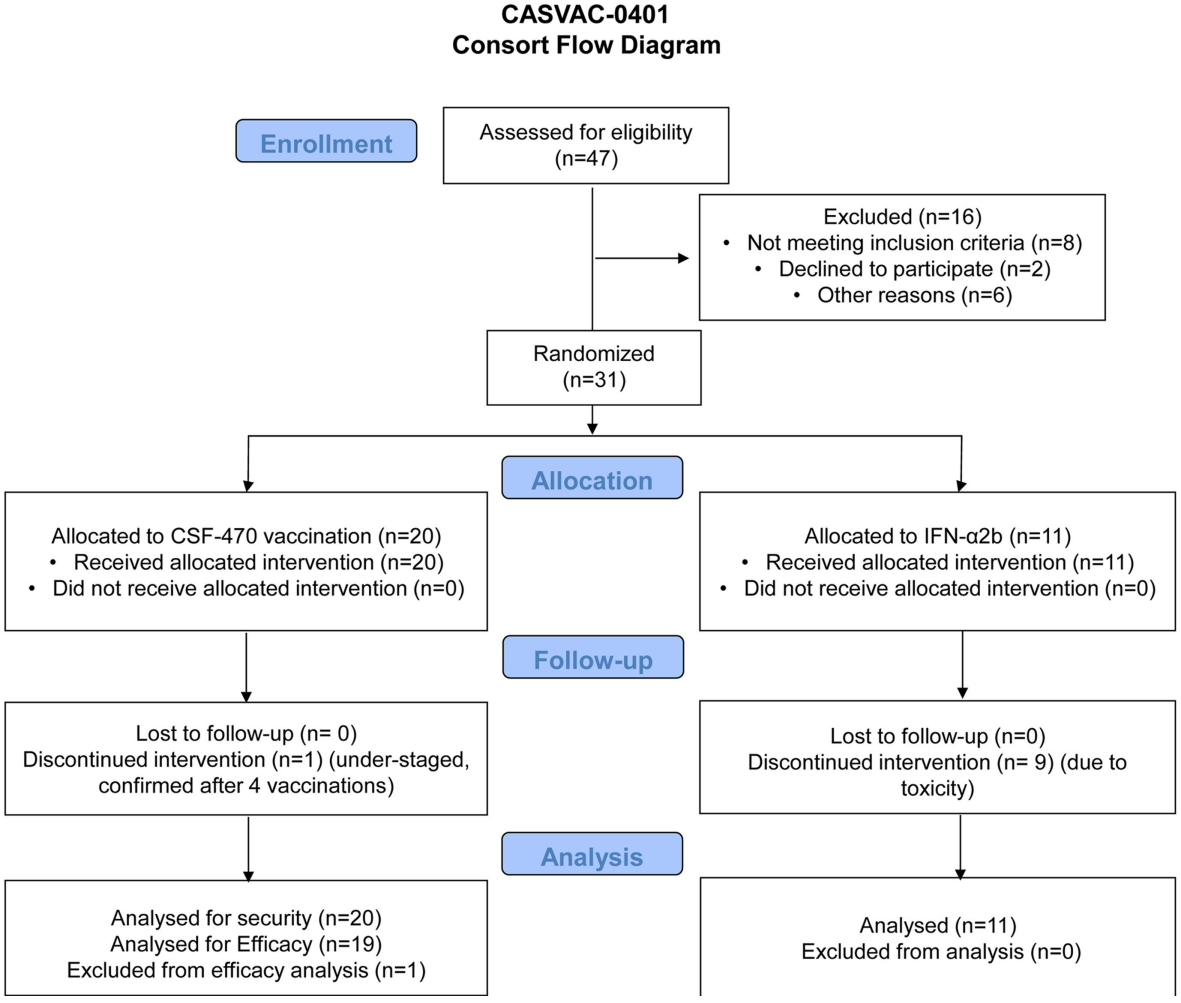
**CASVAC-0401 consort flow diagram**.

The Phase II stage of the CASVAC-0401 study was performed on 31 CM pts who were recruited at the *Instituto Alexander Fleming* between March 2009 and April 2014. The characteristics and evolution of the participating pts are listed in Table 1 and Table S1 in Supplementary Material, in which it can be seen an equitable distribution of pts in both arms of treatment with regard to age, sex, and AJCC-UICC stage stratification. However, primary tumor ulceration was more frequent in the vaccine arm. A Consort Flow chart is shown in Figure 3 describing pts demography and treatment assignment. Twenty-one pts (70%) had the BRAF^V600E^ mutation. Pt #030 was withdrawn from protocol after receiving four vaccines due to confirmation that millimetric lung lesions detected by CT-scan at screening, and informed as non-specific, later developed as overt metastases; the pt was, therefore, at stage IV and understaged at screening. This pt was included in the AEs record but excluded from efficacy calculation.

**Table 1:** **Characteristics of patients (pts) participating in the CASVAC-0401 study**.

Characteristics		Treatment arm		
		CSF-470		IFN-α2b
*n*		20		11

**Sex^a^**				
Male		12 (60.0%)		5 (45.5%)
Female		8 (40.0%)		6 (54.5%)
Age^a^ (median, range)		46.0 (33–61)		53.0 (29–64)
**AJCC-UICC melanoma staging^a^**				
II		2 (10.5%)		0 (0%)
IIB		0		0
IIC		2 (100%)		0
III		17 (89.5%)		11 (100%)
IIIA		3/17 (17.6%)		3/11 (27.2%)
IIIB		8/17 (47.1%)		2/11 (18.2%)
IIIC		4/17 (23.6%)		4/11 (36.4%)
III (Tx^b^)		2/17 (11.7%)		2/11 (18.2%)
**Primary tumors^a^**				
Breslow thickness (median, range)		3.2 (0.89–5.5)		3.0 (0.6–15.0)
Ulceration		12/17 (70.6%)		3/11 (27.3%)
**Treatment**				
Number of vaccine doses received	13	13 (65.0%)	Pts who completed IFN-α2b treatment	2/11 (18.2%)
	12	1 (5.0%)		
	9	1 (5.0%)		
	7	2 (10.0%)		
	6	1 (5.0%)		
	5	1 (5.0%)		
	4	1 (5.0%)		

*^a^No significant differences among CSF-470 and IFNα2b arms were found regarding sex (χ^2^ test, *p* = 0.4362); age (unpaired *t*-test with Welch’s correction, *p* = 0.1990); AJCC-UICC staging (II vs III, χ^2^ test, *p* = 0.2905; IIIA vs IIIB vs IIIC, χ^2^ test, *p* = 0.3263); Breslow thickness (unpaired *t*-test with Welch’s correction, *p* = 0.3253) or ulceration of primary tumors (χ^2^ test, *p* = 0.6550)*.

*^b^Tx, unknown primary tumor*.

### Clinical Response

**Figure 4:**
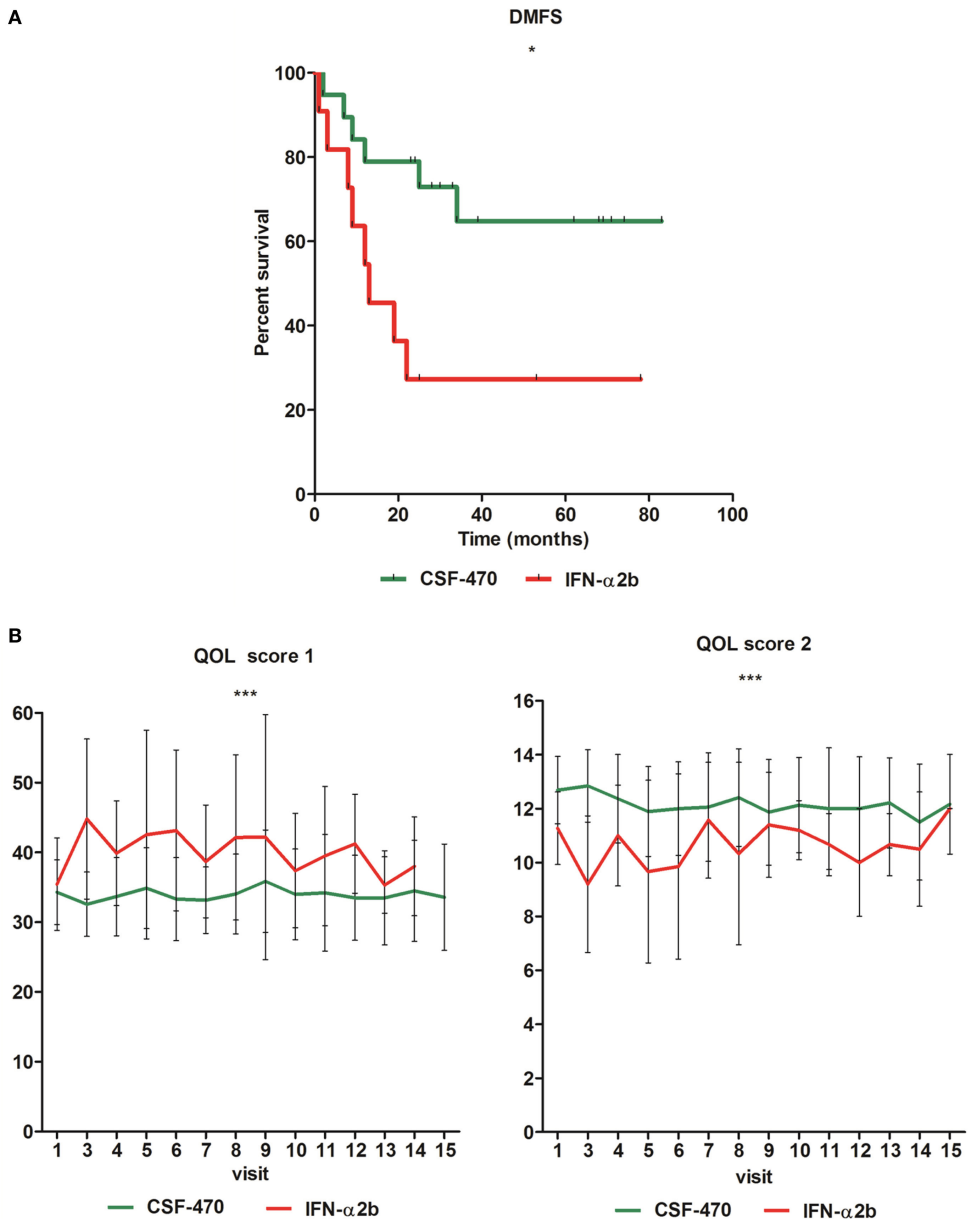
Distant metastasis-free survival (DMFS) of eligible patients (pts) participating in the CASVAC-0401 study (*p = 0.022, Gehan–Breslow–Wilcoxon test) (A). Quality of life (QOL) in all eligible pts participating in CASVAC-0401, evaluated by the European Organization for Research and Treatment of Cancer—Quality of Life Questionnaire (“EORTC QLQ-C30, v 3.0”). Mean and SD are shown (score 1 ***p < 0.0001; score 2 ***p < 0.0001). Score 1 = a higher value implies a poorer QOL; score 2 = a lower value implies a poorer QOL **(B)**.

As of March 2016, pts from CSF-470 vaccine arm received a total of 219 vaccinations (Table 1). A total of 6/19 pts from the vaccine arm and 8/11 pts from the IFN-α2b arm were withdrawn from protocol due to appearance of distant metastases. With a mean and maximum F-UP of 39.4 and 83 months, respectively, DMFS was significantly higher for CSF-470 vaccinated pts than for IFN-α2b pts (*p* = 0.022). DMFS proportion at 25 months was 72.8% for the vaccine arm and 27.2% for IFN-α2b arm (Figure 4A and Table S1 in Supplementary Material). For vaccinated pts, the absolute risk reduction was 51.7% (95%, CI: 19.6–83.7%) and necessary number to treat was 2 (95%, CI: 1–5) at 24 months. However, when both regional and distant progressions were considered (DFS), the difference was not statistically significant as estimated by Kaplan–Meier (*p* = 0.12) (data not shown). Median OS has not been reached for pts in the vaccine arm and it was 39 months for IFN-α2b pts (data not shown).

### AEs and QOL

The AEs are listed in Table 2 (CSF-470-vaccinated pts) and Table 3 (IFN-α2b pts). The most common toxicity in CSF-470 pts (20/20) was an inflammatory reaction at the vaccination site, which were grades 1–2 for most pts. Ninety percentage of the pts presented mild to moderate pain and 40% had pruritus at the vaccination site. In 6/20 pts, the BCG dose had to be reduced by 75% and pts continued vaccination. Systemic AEs were mild (grade 1); one pt with gastritis required treatment with omeprazole and ranitidine and symptoms resolved within 24 h. A total of 4/20 pts presented allergic reactions, mainly cutaneous rash, pruritus, and dyspnea (grades 2–3). In these pts, GM-CSF dose was reduced to 75 μg/day; in only one pt GM-CSF had to be suspended. Fifty milligrams of diphenhydramine were administered p.o. before each vaccination to pts with allergic reactions to prevent symptoms. In the IFN-α2b arm, 3/11 pts interrupted treatment due to excessive toxicity, 5/11 had transitory interruptions and dose reduction and one pt refused to continue treatment within the first month. Only 2/11 pts could complete the 2-year treatment. QOL was significantly better for CSF-470 pts than for IFN-α2b pts as evidenced by both scores obtained from the QOL questionnaire (Figure 4B).

**Table 2:** **AEs in CSF-470-vaccinated patients**.

AE	Patients with AE	Intensity			
		GRADE 1	GRADE 2	GRADE 3	GRADE 4
Inflammatory reaction at vaccine site	20	0	20	0	0
Pain at vaccine site	18	18	0	0	0
Asthenia	16	16	0	0	0
Headache	14	12	2	0	0
Myalgia	9	9	0	0	0
Pruritus at vaccine site	8	8	0	0	0
Abdominal pain	7	7	0	0	0
Diarrhea	6	6	0	0	0
Lumbar pain	6	6	0	0	0
Allergic reaction	4	0	1	3	0
Fever	4	4	0	0	0
Gastritis	4	3	1	0	0
Articular pain	3	3	0	0	0
Fatigue	3	3	0	0	0
Hypotension	3	3	0	0	0
Nausea	3	3	0	0	0
Chills	2	2	0	0	0
Cramps	2	2	0	0	0
Precordial pain	2	2	0	0	0
Vomits	2	2	0	0	0
Anorexia	1	1	0	0	0
Hematospermia	1	1	0	0	0

*AE, adverse event*.

*Total patients receiving at least one dose of vaccine: n = 20*.

**Table 3:** **AEs in IFN-α2b treated patients**.

AE	Patients with AE	Intensity			
		GRADE 1	GRADE 2	GRADE 3	GRADE 4
Asthenia	10	5	5	0	0
Leukopenia	8	4	4	0	0
Neutropenia	7	0	6	1	0
Fever	6	5	0	1	0
Myalgia	6	6	0	0	0
Abdominal pain	5	5	0	0	0
Headache	5	2	3	0	0
Chills	4	3	1	0	0
Alopecía	3	3	0	0	0
Anorexia	3	2	1	0	0
Dry mouth	3	3	0	0	0
Dyspnea	3	3	0	0	0
Hepatic toxicity	3	1	2	0	0
Insomnia	3	2	0	1	0
Lumbar pain	3	3	0	0	0
Articular pain	2	2	0	0	0
Cardiac toxicity	2	1	1	0	0
Cough	2	2	0	0	0
Diarrhea	2	2	0	0	0
Irritability/mood changes	2	2	0	0	0
Nausea	2	2	0	0	0
Allergic reaction at inoculation site	1	1	0	0	0
Anemia	1	1	0	0	0
Bone pain	1	0	1	0	0
Depression	1	1	0	0	0
Dizziness	1	1	0	0	0
Epistaxis	1	1	0	0	0
Erythema	1	1	0	0	0
Fatigue	1	1	0	0	0
Gingival bleeding	1	1	0	0	0
Hypohemoglobinemia	1	0	1	0	0
Hypotension	1	0	1	0	0
Nasal congestion	1	1	0	0	0
Pruritus	1	1	0	0	0
Sensation of ocular pressure	1	1	0	0	0
Somnolence	1	1	0	0	0
Vomits	1	0	1	0	0

*AE, adverse event*.

*Total patients receiving at least one dose of IFN-α2b: n = 11*.

### Monitoring of Vaccine-Induced Immune Responses

**Figure 5:**
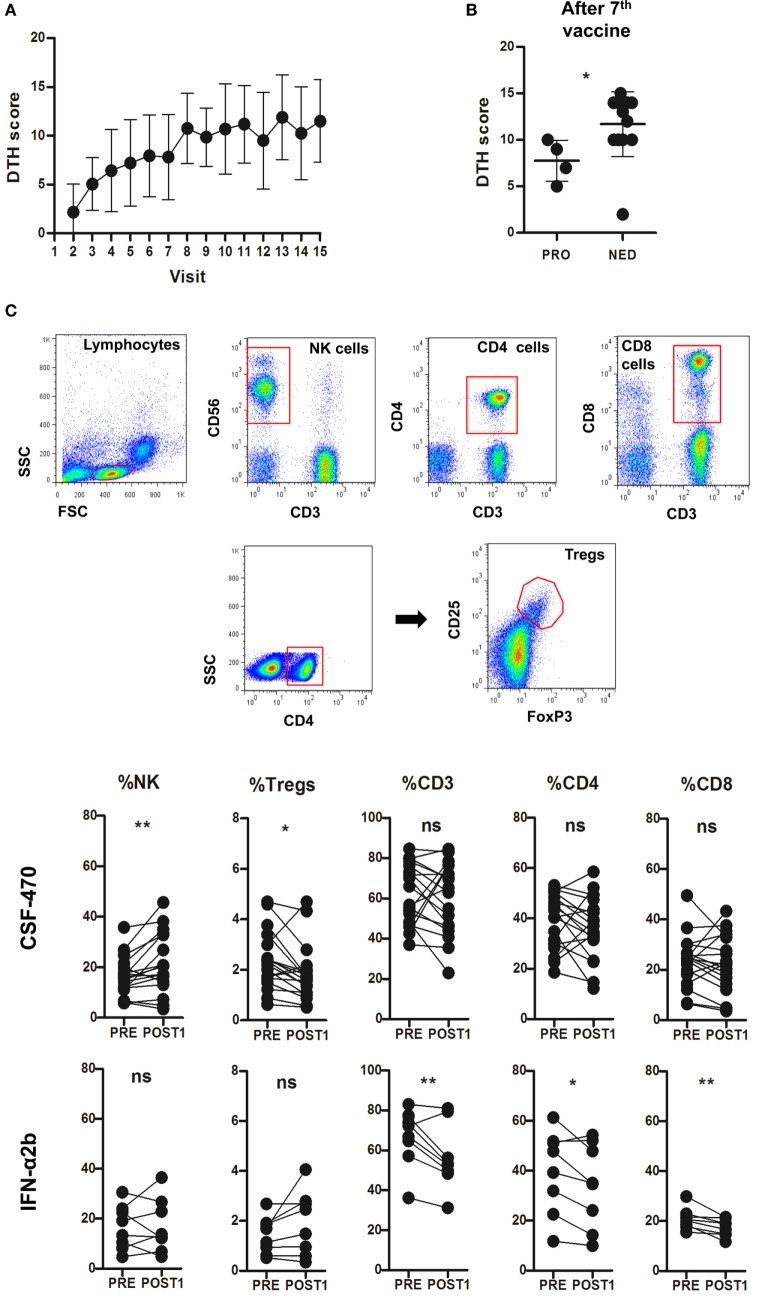
**Mean and SD of delayed-type hypersensitivity (DTH) scores from vaccinated patients (pts) (A)**. Vaccinated pts were grouped according to progressed (PRO) or with no evidence of disease (NED). DTH scores are shown from PRO and NED pts after seven vaccinations (visit 8, *p* = 0.025, Mann–Whitney test) **(B)**. Peripheral blood mononuclear cell immune populations gating strategy and results comparing PRE vs POST1 samples from CSF-470 and IFN-α2b arms. CSF-470: NK PRE vs POST1, **p = 0.008; and Tregs PRE vs POST1, *p = 0.021. IFN-α2b: CD3 PRE vs POST1, **p = 0.0036; CD4 PRE vs POST1, *p = 0.035; and CD8 PRE vs POST1, **p = 0.0075. For all comparisons, paired *t*-test was used. Ns, not significant **(C)**.

Delayed-type hypersensitivity skin test increased in every vaccinated pt after receiving CSF-470 (Figure 5A). After seven vaccinations (visit 8), DTH reactivity was higher in pts with no evidence of disease as compared to those with progressive disease (PRO) (Figure 5B). As for PBMC, population frequencies of PRE and POST1 samples were evaluated. In most CSF-470 pts, only CD3^−^CD56^+^ NK cells significantly increased after vaccination. NK cells from both PRE and POST1 samples from pts receiving vaccine or IFN-α2b were functionally competent, as detected by *in vitro* degranulation assays after co-culturing pts’ PBMCs with K562 or MelC as target cells (*data not shown*). CSF-470 pts had a modest reduction in the CD4^+^CD25^+^FoxP3^+^ Treg lymphocyte population, but no changes in CD3^+^, CD4^+^ and CD8^+^ T cell proportions were observed throughout the vaccination. IFN-α2b treated pts showed a significant decrease in CD3^+^ cell frequency in POST1 samples, particularly in CD4^+^ and CD8^+^ T cells (Figure 5C).

Myeloid suppressor (CD14^+^HLA-DR^−^) cells were less than 1% of CD14^+^ cells in PRE and POST1 samples from all pts (not shown).

**Figure 6:**
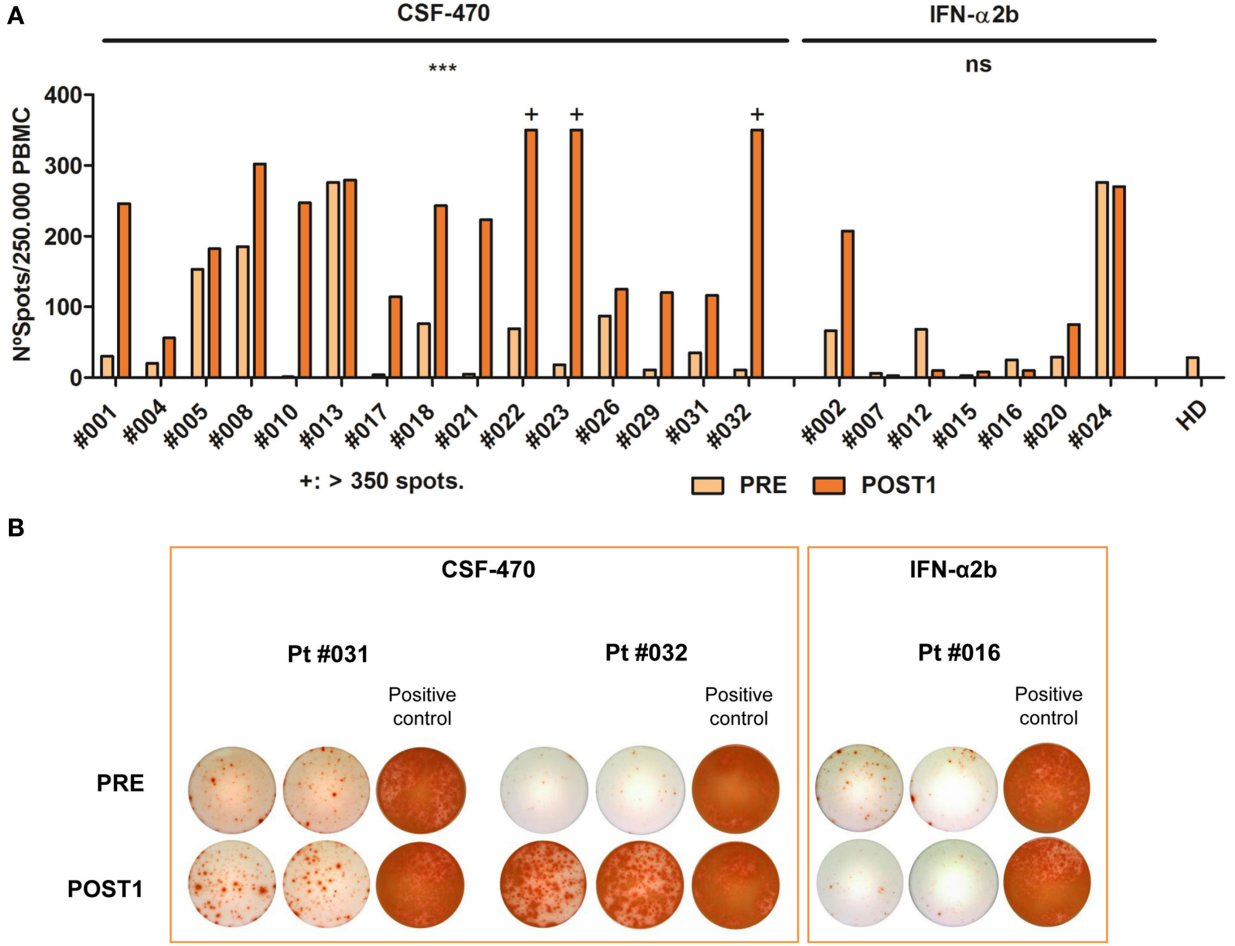
**IFN-γ enzyme-linked immunospot assay (ELISPOT) analysis in PRE and POST1 peripheral blood mononuclear cells (PBMC) samples of patients (pts) participating in CASVAC-0401 study**. **(A)** Each bar corresponds to the mean number of IFN-γ spots quantified for PRE and POST1 PBMC stimulated with vaccine lysate as described in Section “Materials and Methods.” PBMC from a healthy donor (HD) were equally stimulated to set unspecific baseline. Spots could be properly quantified from 1 to 350 spots/well, due to saturation of the signal. CSF-470: PRE vs POST1, ***p = 0.0001, paired *t*-test **(B)** Selected ELISPOT wells are shown to illustrate IFN-γ secreting cells detected after stimulation with CSF-470 lysate from two vaccinated pts and one IFN-α2b-treated pt (duplicates). Positive controls correspond to PBMCs stimulated with anti-CD3 mAb plus PHA as described in Section “Materials and Methods.”

In order to determine peripheral blood T cells reactivity to melanoma Ags in both arms of treatment, IFN-γ ELISPOT analysis was performed after stimulation with a vaccine lysate. PRE and POST1 PBMC samples were analyzed (Figure 6). In the vaccine arm, 15/19 pts could be analyzed. Pt #003 was not analyzed due to early recurrence before obtaining the POST1 sample; PBMC from pt #006 could not be expanded *in vitro* after thawing, pt #011 samples were scarce and in pt #025 blood samples could not be obtained due to bad venous access. In the IFN-α2b arm, 7/11 pts were analyzed. Pt #014, pt #019, pt #027, and pt #028 were not analyzed since their participation in the protocol was discontinued before obtaining the POST1 blood sample. In the vaccine arm, the IFN-γ secreting T cells increased in 14/15 pts and in case of pt #013 they were already high in the PRE sample and remained unchanged in the POST1 sample The difference between IFN-γ secreting T cells in PRE and POST1 samples was statistically highly significant (*p* = 0.0001). Thus, CSF-470 vaccination induced T cell reactivity against melanoma Ags contained in the vaccine cells. On the contrary, in the IFN-α2b arm, in 4/7 pts the number of IFN-γ secreting T cells was low and did not increase with treatment, while in 1/7 they slightly increased. It is remarkable that in pt #002 the number of spots increased with treatment and in pt #024 it was high before treatment and it remained at the same levels at the POST1 sample; both pts remain NED after long F-UP. In the IFN-α2b arm, the difference between the PRE and POST1 samples was not statistically significant (Figure 6).

Thus, there are pts in both arms who recognize Ags contained in CSF-470 vaccine even before any treatment and vaccination induces a significant increase in T cell response.

**Figure 7:**
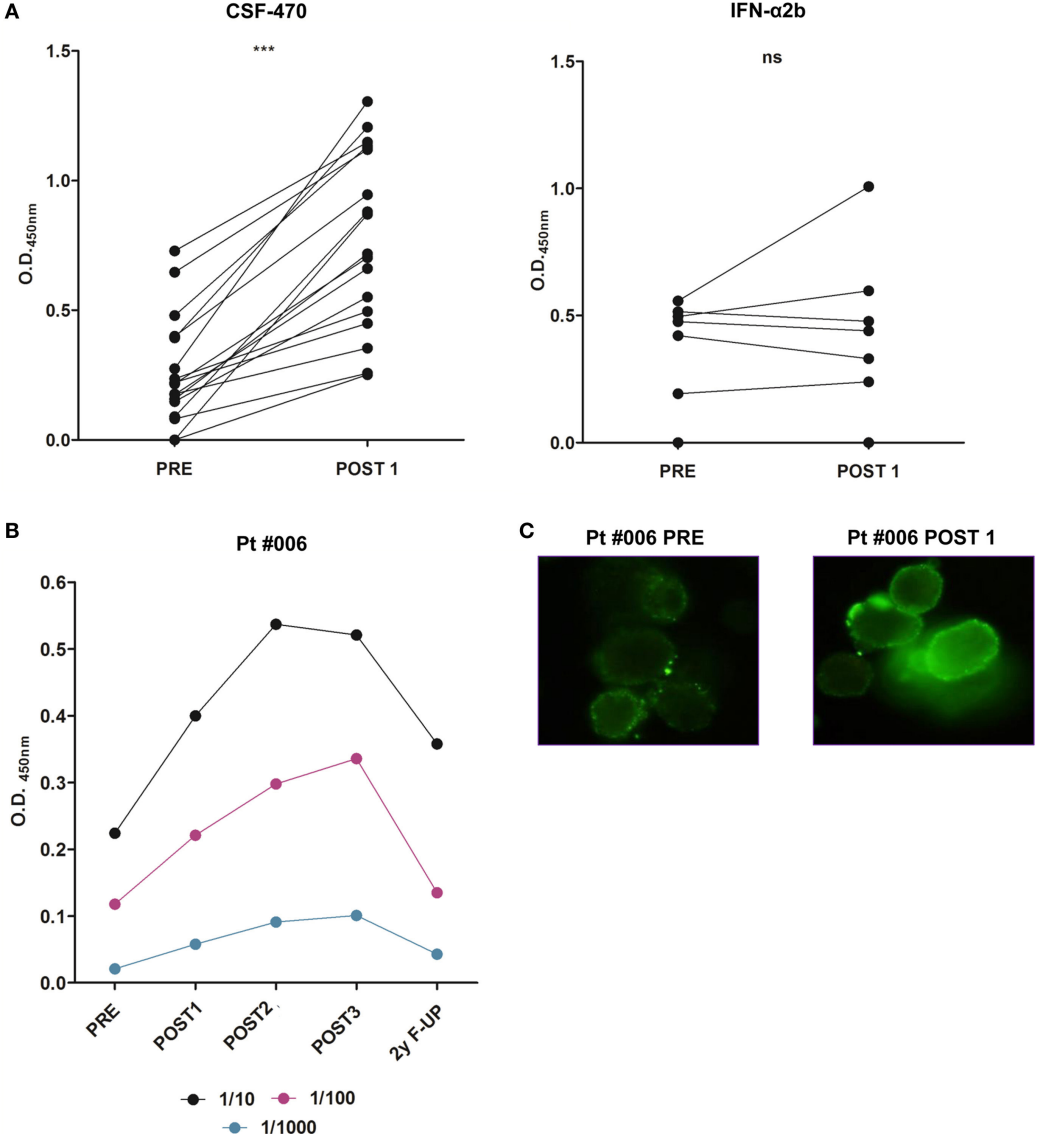
Serum reactivity to MelC of PRE and POST1 samples of patients (pts) participating in CASVAC-0401 study, detected by ELISA (unpaired *t*-test, ***p < 0.0001). Optical density (O.D._450 nm_) from each sample is shown as O.D._450nm_ = O.D. pt serum sample − O.D. healthy donor serum sample. A total of 1/10 serum dilution is shown **(A)**. Serum reactivity to autologous tumor cells of patient #006 was detected by ELISA. A total of 1/10, 1/100, and 1/1,000 serum dilutions are shown **(B)**. Immunofluorescence staining of autologous patient #006 tumor cells with PRE and POST 1 sera showing cell surface reactivity (original magnification: 400×). Antibodies were detected by FITC-rabbit anti IgA, G, and M (DAKO). PRE, POST1, POST2, and POST3 correspond to samples obtained at 0, 6, 12, and 24 months from protocol start. F-UP (follow-up) sample was obtained 24 months after ending protocol participation **(C)**.

All vaccinated pts significantly increased their serum Abs reactivity against MelC (PRE vs POST1 samples) (Figure 7A). In the case of pt #006, increased serum reactivity against autologous tumor cells derived from a s.c. metastasis was detected (Figure 7B); the Abs title returned to the PRE level 2-year after ending her participation in the study (2-year F-UP sample). Particularly, tumor cell surface reactivity was evident in the POST1 sample (Figure 7C).

## Discussion

The adjuvant treatment of stages IIB, IIC, and III CM pts after surgery is a challenging issue. Whereas some untreated pts may remain without recurrence for years, others will relapse within weeks or months. At present, there is no way to differentiate both groups except for risk factors, since present technology is not sensitive enough to detect micrometastatic foci. Since about 30–50% of such pts, according to the disease stage, remain “relapse-free” after surgery without any treatment (43), an equilibrium between effectiveness and lack of toxicity for adjuvant therapy must be found. The main results of the Phase II CASVAC-0401 study are that after a mean F-UP of more than 3 years, 13/19 vaccinated pts (68.4%) remained free of distant metastases, although three of them developed loco-regional s.c. metastases that were removed as permitted by protocol (pts #004, #005, #025). On the contrary, only 3/11 IFN-α2b-treated pts (27%) remained free of distant metastases. The fact that DMFS, but not yet DFS, was statistically higher in the CSF-470 arm than in the IFN-α2b arm is biologically meaningful since it has been shown that pts with resectable loco-regional metastases remain at low risk of further progression (44). Also, the administration of CSF-470 vaccine plus BCG plus rhGM-CSF was safe and well tolerated; it was associated with a better QOL than IFN-α2b treatment. The observed AEs were similar to those reported in a previous Phase I study (29) and were mostly related to local reaction.

As to the mechanism of action, we suggest that CSF-470 plus BCG plus rhGM-CSF triggered both innate and adaptive immunities. CSF-470 is a mixture of four CM cell lines which are heterogeneous for HLA class I haplotype and for expression of melanoma associated Ags (30). With respect to induction of adaptive immunity, we suggest the following mechanism: the danger signals provided by allogeneic HLAs and adjuvants BCG and GM-CSF would force attraction of Ag-presenting cells (DCs and macrophages) to the vaccination site, where they would phagocyte tumor Ags (26) and present them in an adequate HLA setting to naïve lymphocytes, either *in situ* or upon migration to draining lymph nodes (45, 46).

As we have shown in this paper, each cell line displays a characteristic set of shared Ags, and their combination offers the immune system a wide variety of melanoma differentiation Ags, cancer-testis Ags, and gangliosides. We have demonstrated by ELISPOT analysis that CSF-470-vaccinated pts develop strong T cell reactivity against a vaccine lysate. This result suggests that a considerable amount of vaccinated pts’s PBMC are reacting against melanoma Ags contained in the vaccine. The nature of the recognized Ags deserves further analysis. However, it is worth mentioning that some pts in both arms had reactivity against CSF-470 Ags even before treatment. Those pts are long-term survivors, and this suggests that the CSF-470 vaccine is a carrier of relevant Ags and that, if detected before any treatment starts, might be a marker of good prognosis. In most vaccinated pts, the IFN-γ secreting cells increased, demonstrating that this vaccination system induces a Th1 response. The fact that PRE vaccination T cells could be stimulated to secrete IFN-γ upon incubation with CSF-470 lysate implies that an immune response probably directed to common melanoma Ags present in the vaccine cells has been elicited.

Coincidently, we have also observed in this study significant increases in DTH and humoral responses (serum Abs) after CSF-470 vaccination; and it has been extensively demonstrated that DTH is useful to evaluate cellular immune responses (47, 48).

With respect to the innate immunity response, we have observed a significant increase in functional NK cells in PBMC from CSF-470 pts after the sixth vaccination (6 months of treatment), and we suggest that NK cells exert killing of tumor cells that have lost HLA-I expression (49, 50). It is important to note that in 6/14 vaccinated pts amenable to analysis, less than 50% of tumor cells retained HLA expression and that in some pts HLA expression decreased from primary tumors to metastases (Table S1 in Supplementary Material). These findings could lead us to postulate that development of innate immunity may have contributed to the more favorable course of vaccinated pts. Such hypothesis is supported by the experimental work of Malladi et al., who found that ablation of NK cells dramatically increased the ability of latency competent cells to survive and metastasize (51). Since we also observed an increment in serum Abs recognizing autologous tumor cells (pt #006), it may also be suggested that the strong Ab response mediates Ab-dependent cellular cytotoxicity targeting either circulating tumor cells and/or micrometastatic foci.

GM-CSF containing vaccines were previously shown to expand a population of CD14^+^HLA-DR^−^ myeloid cells endowed with a TGF-β-mediated immune suppressive activity at short intervals near vaccine and GM-CSF administration, revealing an immune suppression activity modulated by GM-CSF-based autologous vaccine, as reported by Filipazzi et al. (52). However, we only detected minimal numbers of circulating myeloid suppressor cells either before (PRE) or after 6 months treatment (POST1). Since samples were obtained at fixed times as established by the protocol, our data may not be comparable our results to those previously reported.

Immune checkpoint blockade has the capacity to enhance and sustain endogenous immunity against non-mutated tumor-associated Ags as well as uniquely mutated Ags, establishing durable tumor control (53). After anti-CTLA-4 treatment, evidence of an immune response targeting tumor neoepitopes provided a genetic basis for anti-immune checkpoint response. Mutational load was associated with the degree of clinical benefit, and a neoantigen landscape is specifically present in tumors with a strong response to CTLA-4 blockade. Predicted neoantigens were able to activate T cells from the pts treated with ipilimumab *in vitro* (54). Also, inhibition of PD1/PD-L1 pathway was shown to influence the evolving landscape of tumor neoantigens during the emergence of acquired resistance to treatment, involving genetic changes in the tumor to eliminate such neoantigens (55). Given the safety and good tolerance of CSF-470 vaccine and the results obtained so far in this Phase II study, it would be interesting to test if addition of immune checkpoint inhibitors to CSF-470 vaccination could result in better stimulation of immune responses and higher clinical benefit to high risk CM pts in a future study.

Expressed somatic mutations present in the CSF-470 vaccine will be identified after whole exome sequencing of the CM cell lines; *in silico* epitope prediction will be performed and epitope-specific peptides will be selected to analyze their immune recognition by vaccinated pt’s T cells, considering their HLA restriction. Also, melanoma biopsies from some pts will be equally analyzed in the near future to investigate the epitope spreading hypothesis after CSF-470 vaccination.

To summarize, the CSF-470 vaccine plus BCG plus rhGM-CSF administered as adjuvant therapy to stages IIB, IIC, and III CM pts was superior to IFN-α2b treatment in delaying or preventing progression to distant metastasis; it was well tolerated and it was associated with an improved QOL. These results encourage the continuation of a Phase III part of the CASVAC-0401 study.

## Ethics Statement

This study was carried out in accordance with the recommendations of the “Ethics Committee of the Instituto Médico Especializado Alexander Fleming” with written informed consent from all subjects. All subjects gave written informed consent in accordance with the Declaration of Helsinki. The protocol was approved by the “Ethics Committee of the Instituto Médico Especializado Alexander Fleming (Buenos Aires, Argentina), and by the local Regulatory Agency (ANMAT - Argentina).” The “Ethics Committee of the Instituto Médico Especializado Alexander Fleming (Buenos Aires, Argentina)” is reputed by the Central Ethics Committee of the City of Buenos Aires (Argentina).

## Author Contributions

JM: conception and design of the study; collection and assembly of data; data analysis and interpretation; and manuscript writing. JM was the principal investigator of the study. MP, MA, and PB: collection and assembly of data; data analysis and interpretation; and manuscript writing. ML: conception and design of the study; data analysis and interpretation; and manuscript writing. SM, MC, AB, AO, FR, EE, EL, and MB: data analysis and interpretation and manuscript writing. JO: collection and assembly of data; data analysis and interpretation; and manuscript writing.

## Conflict of Interest Statement

ML is Advisor of Laboratorio Pablo Cassará. The other authors declare no conflict of interests. The reviewer VR and handling Editor declared their shared affiliation, and the handling Editor states that the process nevertheless met the standards of a fair and objective review.

## Abbreviations

Ab, antibodies; Ags, antigens; BCG, Bacillus Calmette–Guerin; CM, cutaneous melanoma; DCs, dendritic cells; DFS, disease-free survival; DMFS, distant metastasis-free survival; DTH, delayed-type hypersensitivity; FFPE, formalin-fixed, paraffin-embedded; F-UP, follow-up; HD, healthy donor, HLA, human leukocyte antigen; Igs, immunoglobulins; MAbs, monoclonal antibodies; MU, million units; NED, no evidence of disease; OS, overall survival; pts, patients; PBMC, peripheral blood mononuclear cells; PBS, phosphate saline buffer; PI, proliferative index; PRO, progressive disease; QOL, quality of life; rhGM-CSF, recombinant human granulocyte macrophage-colony stimulating factor.
