# Supplementary material for: Phase II Study of Adjuvant Immunotherapy with the CSF-470 Vaccine Plus Bacillus Calmette–Guerin Plus Recombinant Human Granulocyte Macrophage-Colony Stimulating Factor vs Medium-Dose Interferon Alpha 2B in Stages IIB, IIC, and III Cutaneous Melanoma Patients: A Single Institution, Randomized Study
Source: Front Immunol. 2017 May 31;8:625. doi: 10.3389/fimmu.2017.00625 (PMC5449770; doi:10.3389/fimmu.2017.00625)
Supplement: Supplementary file 1 [file Table_1.PDF]

Supplementary Table 1: Characteristics and evolution of participating patients.

Patient	Sex	Arm	Age	AJCC-UICC staging	Pathologic staging		BRAF status	Primary tumors						Metastases					DMFS	DFS	OS
					Tis	NO		Breslow thickness (mm)	U	PI (%)	HLA-I (%)	LI	S	Nodal status	R	LR	PI (%)	HLA-I (%)			
**001**	F	CSF-470	33	IIC	T4b	-	V600E	4.1	yes	0,51	nd	NB	no	-	-	-	-	-	34	34	47
**003**	M	CSF-470	42	IIIC	T4b	N2b	V600E	5	yes	44.2%	>75%	NB	yes	1m 2M	no	-	na	na	2	2	12
**004**	F	CSF-470	41	IIIC	T3b	N2b	V600E	2.3	yes	>25%	>75%	NB	no	1m 2M	yes	-	44.3%	>75%	74+	6	74+
**005**	F	CSF-470	33	IIIC	T1b	N2c	V600E	0.89	yes	0	nd	B	no	-	-	+	38.8%	>50%	68+	3	68+
**006**	F	CSF-470	51	IIIB	T3b	N1a	V600E	3	yes	13.1%	>50%	NB	no	1m	no	-	na	na	25	25	58+
**008**	M	CSF470	52	IIIB	T2a	N2b	V600E	1.2	no	65.1%	<50%	NB	no	1m 2M	yes	-	23.4%	<50%	83+	83+	83+
**010**	M	CSF-470	48	IIIB	T4b	N1a	V600E	4.1	yes	33.2%	>50%	NB	no	1m	no	-	33.4%	>50%	71+	71+	71+
**011**	M	CSF-470	61	III	Tx	N2b	nd	*unknown*						2m 1M	yes	-	22.6%	na	69+	69+	69+
**013**	M	CSF-470	50	IIIA	T3a	N1a	V600E	4	no	>25%	na	NB	no	1m	no	-	50.3%	nd	62+	62+	62+
**017**	M	CSF-470	47	IIIA	T3a	N1a	V600E	3	no	25.2%	>75%	NB	no	1m	no	-	21.9%	>75%	39+	39+	39+
**018**	F	CSF-470	39	IIIB	T2b	N2a	V600E	2	yes	20.2%	<50%	NB	no	2m	no	-	50.9%	<50%	12	12	30
**021**	F	CSF-470	51	IIIB	T4b	N1a	V600E	5.5	yes	0,5	<50%	NB	no	1m	no	-	21.0%	<50%	30+	30+	30+
**022**	M	CSF-470	46	IIIB	T3b	N2a	V600E	2.25	yes	34.5%	>75%	B	no	2m	yes	-	42.6%	>75%	34+	34+	34+
**023**	M	CSF-470	35	IIIA	T3a	N1a	WT	4	no	8.5%	<50%	NB	no	1m	no	-	na	na	33+	33+	33+
**025**	F	CSF-470	61	III	Tx	N2c	WT	*unknown*						-	-	+	5.0%	nd	28+	28+	28+
**026**	M	CSF-470	40	IIIC	T3b	N3	V600E	3.2	yes	36.7%	>75%	NB	no	9m 1M	yes	-	51.0%	<50%	9	5	14
**029**	M	CSF-470	35	IIIB	T3b	N1b	V600E	4	yes	49.8%	>75%	NB	yes	1M	yes	-	55.0%	>50%	7	7	14
**031**	M	CSF-470	46	IIC	T4b	-	WT	4.2	yes	40.8%	<50%	NB	no	-	-	-	-	-	23+	23+	23+
**032**	F	CSF-470	43	IIIB	T2a	N2c	WT	2	no	28.8%	<50%	NB	no	-	no	+	21.9%	<25%	24+	24+	24+
**002**	F	IFN-α2b	37	IIIC	Tx	N3	nd	*unknown*						1m 1M	yes	+	36.6%	nd	78+	78+	78+
**007**	F	IFN-α2b	45	IIIA	T1a	N2a	V600E	0.6	no	26.5%	>75%	B	no	2m	no	-	na	na	53+	53+	53+
**012**	F	IFN-α2b	41	IIIC	Tx	N3	V600E	*unknown*						massive	yes	-	38.0%	<50%	9	9	17
**014**	M	IFN-α2b	64	IIIA	T3a	N1a	WT	2.5	no	>25%	na	NB	no	1m	no	-	na	na	19	19	39
**015**	M	IFN-α2b	54	IIIB	T4b	N1b	nd	15	yes	>25%	na	NB	no	1M	no	-	na	na	8	8	26
**016**	F	IFN-α2b	57	III	Tx	N2b	V600E	*unknown*						1m 1M	yes	-	16.5%	<50%	12	12	48+
**019**	M	IFN-α2b	64	III	Tx	N1b	V600E	*na*						1M	yes	-	63.2%	<50%	3	3	26
**020**	M	IFN-α2b	51	IIIA	T2a	N2a	WT	1.2	no	28.8%	>75%	NB	no	2m	yes	-	48.6%	>50%	22	22	34+
**024**	F	IFN-α2b	29	IIIB	T3b	N1b	V600E	3.5	yes	24.3%	<50%	NB	no	1M	no	-	na	na	25+	25+	25+
**027**	F	IFN-α2b	56	IIIC	T4b	N3	V600E	14.5	yes	38.2%	>75%	NB	yes	massive	yes	+	48.0%	>75%	13	3	20
**028**	M	IFN-α2b	53	IIIC	Tx	N3	V600E	*na*						massive	yes	-	50.0%	<25%	1	1	4

Staging was assigned according to the AJCC-UICC criteria [26], regarding clinical and pathologic staging of primary tumors (Tis) and metastases (NO). When unknown primary tumor (Tx), pathologic staging was assigned only when NO=N3. *Sex: F=female, M=male; BRAF status:* WT= wild type; U= Ulceration; PI= Proliferative Index; HLA-I: positive tumor cells (%); *Lymphocyte infiltrate (LI):* NB= non-brisk, B= brisk; S= satellitosis; R= capsular rupture; *Nodal status:* M= macrometastases, massive= multiple nodal metastases, m= micrometastases; LR= Loco-Regional metastases; DMFS= Distant metastases-free survival; DFS= Disease-free survival; OS= Overall Survival; nd= not determined; na= not available.
