# Supplementary material for: Phase II Study of Adjuvant Immunotherapy with the CSF-470 Vaccine Plus Bacillus Calmette–Guerin Plus Recombinant Human Granulocyte Macrophage-Colony Stimulating Factor vs Medium-Dose Interferon Alpha 2B in Stages IIB, IIC, and III Cutaneous Melanoma Patients: A Single Institution, Randomized Study
Source: Front Immunol. 2017 May 31;8:625. doi: 10.3389/fimmu.2017.00625 (PMC5449770; doi:10.3389/fimmu.2017.00625)
Supplement: Supplementary file 2 [file Data_Sheet_1.DOCX]

**Supplementary data sheet 1: CASVAC-0401 Protocol information**

**CASVAC-0401 Protocol**: randomized, comparative phase 2/3 study between treatment with CSF-470 vaccine (allogeneic, irradiated), plus BCG and Molgramostim (rhGM-CSF) as adjuvants, and interferon-alfa 2b (IFN-α2b), in stages IIB, IIC and III post-surgery cutaneous melanoma patients.

**Purpose:** Randomized, open, Phase 2-3 study, comparative between treatment with CSF-470 vaccine (allogeneic, irradiated), with BCG and Molgramostim (rHuGM-CSF) as adjuvants, and interferon alfa 2b (IFN-α2b) treatment , in stages IIB, IIC o III (AJCC) post-surgery cutaneous melanoma patients.

This study has been approved by “Administración Nacional de Medicamentos, Alimentos y Tecnología Médica” (ANMAT, Argentina), number 1299/09 (www.anmat.gov.ar).

This study is registered in ClinicalTials.gov (ClinicalTrials.gov identifier: NCT01729663).

The study Institution is Instituto Médico Especializado Alexander Fleming, Buenos Aires, Argentina. The Sponsor is Laboratorio Pablo Cassará (LPC, S.R.L.).

**Selection of patients**:

Dr. José Mordoh was the Principal Investigator of the study. Study population include a total of 108 patients with a 2:1 ratio vaccine : IFN-α2b (72 patients will receive CSF-470 vaccine and 36 patients will receive alpha IFN-alpha 2b) for a total of 24 months. The population size of the study was calculated based on the results of the phase I study of Vaccimel + BCG + GM-CSF (1), in which DFS at 2 years was estimated to be 84% for vaccinated pts and considering a DFS of 53% for IFN-α2b treated pts estimated from the ECOG1684 study (2), accepting an alpha risk of 0.05 and a beta risk of 0.2 in a two-sided test, an anticipated drop-out rate of 30% using, ARCSINUS approximation (GRANMO calculator, Institut Municipal d'Investigació Mèdica, Barcelona, Spain)

Ages Eligible for Study: 18 Years to 65 Years

Genders: Both

***Inclusion Criteria:***

- Histologically confirmed cutaneous melanoma stages IIB, IIC or III (AJCC) patients non-detectable disease (NED) after surgery as asserted by CAT scans. Melanoma pts with unknown primary tumor site could be included in the study.
- Life expectancy > 6 months.
- Performance status (ECOG) 0 or 1.
- Time post-surgery not more than 4 months.
- No chemotherapy, radiotherapy or any biological treatments prior to this study.
- Patients must have CAT scans of brain, thorax, abdomen and pelvis in the 60 previous days to trial enrolling.
- To have laboratory studies (no more than 15 days before entering the study).
- Laboratory eligibility criteria include: hematocrit: ≥35 (hemoglobin > 10,5 gr %); WBC count > 3500/mm^3^, platelets > 100.000/mm^3^, total and direct bilirubin, serum oxalacetic transaminase and glutamic pyruvic transaminase < 1.5 fold the upper normal value; LDH ≤ 450 mU/ml.
- Negative serology for HIV, anti-HCV and HBsAg.
- Serum creatinine < 2.0 mg%
- All patients must give written informed consent before inclusion in the Study.

***Exclusion Criteria:***

- Pregnant or breast-feeding women.
- Diabetes (Type I or II).
- Antecedents of psychiatric diseases.
- Evidence of active infections.
- Antecedents of viral or autoimmune hepatitis.
- Previous autoimmune diseases.
- Morbid Obesity, defined as CMI (Corporal mass index)>37 kg/m2 in women and >40 kg/m2 in men.
- Other diseases that require treatment with regular corticoids or non-steroids anti-inflammatory drugs.

**Scheme and treatment plan, including administration schedule:** CSF-470 treatment will consist of four vaccine doses id injection (three weeks apart), then one dose every two months for the first year and them every three months for the second year. Each participant will attend a total of 15 visits and will receive 13 vaccines along the 2-year protocol.

Each vaccine consist of a mixture of 17,6x10^6^ melanoma cells, from four melanoma cell lines, not genetically modified and lethally irradiated. As adjuvants, BCG (120 µg prot) the first day and rHuGM-CSF (Molgramostim, 400 µg, fractionated in four days doses) will be used.

IFN-alpha 2b treatment will consist of s.c. injection of 10 million units (MU) (5 t/w) for four weeks and then 5 MU (3t/w) for 23 months.

- **Rules for dose modification:**

**BCG:** In case of an excessive reaction after application of BCG with the vaccination the dose will be reduced to 25% of the planned dose. If the patient continues to develop significant toxicity, the dose of BCG will be reduced to 5%. Continuing the marked toxicity BCG will not be given in the following vaccinations.

**GM-CSF:** In case of a severe reaction after the first dose of rHuGM -CSF and not resolved with analgesics and rest in the supine position, such as severe allergic reactions grade 3-4, grade 3 -4 chest pain, muscle or bone pain grade 3-4, the dose of HuGM -CSF will be reduced to 75 µg/day. In these patients administration of anti-histamines (diphenhydramine) before each vaccination reduces the severity off allergic reactions. If these symptoms persist, it will stop the Molgramostim.

**-Measurement including effect of treatment response criteria, definitions of response and survival, and methods of measurement**

*Overall survival (OS):* The survival time is defined as the time from the date the patient signs the Informed Consent patient to the date of death from any cause.

*Disease-free Survival (DFS):* The time from the date of signing the Informed Consent and time of discovery of disease progression. Disease progression is considered a ≥ 25% increase in the tumor mass compared with nadir on two consecutive observations separated by ≥ 4 weeks.

Diagnosis of disease progression will be based on history, physical examination of the patient and the results obtained in complementary examinations to be conducted in a systematic way during the protocol. The following complementary studies will be performed during the development of protocol:

-Chest x-ray (front and profile) to be held at visit 5 (corresponding to the week 10 of treatment, day 63 of the study); every 2 months from the day 123 study (corresponding to week 18 of treatment - visit 6) during the first year of treatment; and every 3 months (from week 62 of treatment, corresponding to Visit 11) during the second year.

- Ultrasound of the abdomen and pelvis to be held at visit 5 (corresponding to the week 10 of treatment (day 63 of the study); every 2 months from the day 123 study (corresponding to week 18 of treatment - visit 6) during the first year treatment; and every 3 months (from week 62 of treatment, corresponding to Visit 11) during the second year.

-CT brain, chest, abdomen and pelvis which are held annually, to meet the first and second year of treatment (14 visits 10 and 15 respectively).

Importantly, occurrence of distant metastases is a criterion for patient withdrawal from protocol, unless such metastases they can be surgically removed, or if there were not a better therapeutic treatment to offer to the patient. In such cases, although it was considered the onset of these metastases as a sign of disease progression, the patient will not be withdrawn from the study. These patients will be included in the analysis of OS, but separately from the rest of the population analyzed. During the course of the study biopsies will be analyzed to assess the evolution of the disease and how the patient is responding to treatment.

*Distant metastasis free survival (DMFS):* is defined as the elapsed time from the date the patient signs the informed consent until evidence of distant progression, defined as the appearance of visceral metastases, or non-locoregional dermal or lymph node metastases.

*Partial or Complete Remission:* Since this protocol are used for increasing the response immune against tumors, the settings will be taken into account parameters for the immune response to activity in solid tumors (IRRC). In those patients who experienced disease progression but to fulfill with features previously mentioned, they have continued in the protocol, evaluate whether to continue treatment study presented a partial remission or full of metastatic lesions. Partial remission was considered a decrease ≥ 50% of the tumor load from baseline on two consecutive observations separated for ≥ 4 weeks.

Complete remission was considered the disappearance of all lesions on two observations row separated by an interval ≥ 4 weeks.

*Stable disease*: It is defined as the situation in which neither sufficient decrease ≥ 50% of the tumor load from baseline on two consecutive observations separated for ≥ 4 weeks or an increase of 25 % compared with nadir is observed.

**- Reasons for early cessation of trial therapy:**

- Voluntary retirement
- Protocol violations
- Pregnancy
- Development of a viral hepatitis B or C or HIV infection during the established protocol.
- Disease progression requiring the institution of another therapy, except metastases that could be surgically removed or loco-regional.
- Steroid requirement.
- Severe allergic reactions.
- Receiving any other treatment for the melanoma, during the course of the protocol.
- Present treatment toxicity requiring the suspension.

**- Objectives and entire statistical section (including endpoints**):

Both treatments will compare:

- *Toxicity*

Clinical manifestations

Laboratory abnormalities

- *Effectiveness*

Overall survival; Disease-Free survival, Disease-free survival at a distance, stable disease; partial or complete remission.

- *Immune response*

Cellular immunity (DTH only in vaccinated patients)

humoral immunity

- *Quality of life*

EORTC QLQ - C30

ECOG Performance status

**Statistical analysis:**

*Toxicity:* Incidence of each adverse event based on their severity and relationship to study treatment for each treatment group.

*Effectiveness:* Overall survival , disease-free survival and disease-free survival and distant disease stable Kaplan - Meier method with a log- rank test 2-tailed to estimate the difference between the groups, with a significance level of 0 05 .

*Quality of life:* Evaluated by EORTC QLQ - C30 and ECOG Performance status - Multivariate comparison.

**References**

1. Barrio MM, de Motta PT, Kaplan J, von Euw EM, Bravo AI, Chacón RD, Mordoh J. A phase I study of an allogeneic cell vaccine (VACCIMEL) with GM-CSF in melanoma patients. *J Immunother* (2006) **29**:444–54. doi:10.1097/01.cji.0000208258.79005.5f

2. Kirkwood JM, Strawderman MH, Ernstoff MS, Smith TJ, Borden EC, Blum RH. Interferon alfa-2b adjuvant therapy of high-risk resected cutaneous melanoma: the Eastern Cooperative Oncology Group Trial EST 1684. *J Clin Oncol* (1996) **14**:7–17.
